# Draft Genome Sequences of 40 Pathogenic *Leptospira* Strains Isolated from Cattle in Uruguay

**DOI:** 10.1128/MRA.00893-19

**Published:** 2019-11-21

**Authors:** Cecilia Nieves, Ignacio Ferrés, Florencia Díaz-Viraqué, Alejandro Buschiazzo, Leticia Zarantonelli, Gregorio Iraola

**Affiliations:** a Laboratorio de Microbiología Molecular y Estructural, Institut Pasteur de Montevideo, Montevideo, Uruguay; b Unidad Mixta Pasteur + INIA (UMPI), Institut Pasteur de Montevideo, Montevideo, Uruguay; c Bacterial Symbionts Evolution, INRS-Institut Armand-Frappier Laval, Quebec, Canada; d Microbial Genomics Laboratory, Institut Pasteur de Montevideo, Montevideo, Uruguay; e Host-Parasite Interactions Laboratory, Institut Pasteur de Montevideo, Montevideo, Uruguay; f Joint International Unit “Integrative Microbiology of Zoonotic Agents” IMiZA, Institut Pasteur de Montevideo, Montevideo, Uruguay; g Joint International Unit “Integrative Microbiology of Zoonotic Agents” IMiZA, Institut Pasteur, Paris, France; h Département de Microbiologie, Institut Pasteur, Paris, France; i Wellcome Sanger Institute, Hinxton, United Kingdom; j Center for Integrative Biology, Universidad Mayor, Santiago de Chile, Chile; University of Arizona

## Abstract

Pathogenic *Leptospira* species represent a major concern for livestock but also for human health, as they cause zoonotic infections. Forty strains representing L. interrogans, L. borgpetersenii, and L. noguchii were isolated from naturally infected cattle in Uruguay. Here, we report the whole-genome sequences for these strains.

## ANNOUNCEMENT

*Leptospira* is a bacterial genus whose taxonomic diversity has recently scaled up to over 60 described species, which can be grouped into four main phylogenetic lineages (1–3). Historically, these lineages were classified as “pathogens,” “intermediates,” and “saprophytes.” The pathogens (and intermediates to a lesser extent) are the recognized causative agents of human leptospirosis, an emerging zoonotic disease that accounts for more than 1 million severe cases and over 60,000 deaths every year worldwide (4). In cattle, leptospirosis causes reproductive problems, including abortions and stillbirths, constituting an important concern for livestock health and industries (5). In Uruguay, serologic studies of animals have historically shown high prevalence of exposure to *Leptospira*, with individual seropositivity ranging from 25 to 50% and herd prevalence from 50 to 70% (6, 7). In a recent study, we described the isolation, serotyping, and molecular characterization of 40 autochthonous pathogenic *Leptospira* strains recovered from cattle (7). Here, we report the whole-genome sequences and main genomic features of these strains.

*Leptospira* isolates were cultured in Ellinghausen-McCullough-Johnson-Harris (EMJH) medium supplemented with 100 μg/ml 5-fluorouracil at 29°C and observed under dark-field microscopy weekly for up to 6 months. In case of contamination, cultures were filtrated through a 0.22-μm sterile syringe filter and subcultured in fresh EMJH medium without 5-fluorouracil (6). Genomic DNA was purified with the PureLink genomic DNA minikit (Invitrogen). Whole-genome sequencing libraries were prepared with Nextera XT, and sequencing was performed using Illumina technology to produce paired-end reads (2 × 150 cycles) at the P2M core facility (Mutualized Platform for Microbiology) in the Institut Pasteur (Paris, France) or at the sequencing facility in the Institut Pasteur de Montevideo (Montevideo, Uruguay). Raw reads were quality filtered, assembled, and annotated as previously described (8). Species membership was confirmed by inspecting the 16S rRNA gene sequence and by calculating the average nucleotide identity (ANI) using in-house R scripts against public reference *Leptospira* genomes. In all cases, species coincided with previous determinations using molecular methods, representing 21 L. interrogans, 11 L. borgpetersenii, and 8 L. noguchii strains. For L. interrogans, the *N*_50_ value ranged from 10,222 to 49,707 bp, and the genome size ranged from 3.96 to 4.62 Mb. For L. borgpetersenii, the *N*_50_ value ranged from 31,998 to 44,974 bp, and the genome size, from 3.67 to 3.74 Mb. For *L. noguchii*, the *N*_50_ value ranged from 9,345 to 33,876 bp, and the genome size, from 3.86 to 4.75 Mb. More details on the assembly and annotation summary statistics are available at https://doi.org/10.6084/m9.figshare.8277623.v3.

An initial characterization of these strains obtained by recovering multilocus sequence typing (MLST) genes (scheme 3 available at PubMLST) from whole-genome sequences revealed that the L. interrogans strains were genetically monomorphic at this level, being assigned to the sequence type 58 (ST-58). In contrast, all of the L. borgpetersenii and *L. noguchii* strains harbored new combinations of previously described alleles or novel alleles representing new genotypes. This genomic data set represents the diversity of *Leptospira* species circulating in naturally infected cattle in Uruguay. The in-depth comparative analysis of these genomes together with those from strains isolated from humans and livestock will allow us to precisely determine epidemiological features of pathogenic *Leptospira* in this geographic region and improve our understanding of the genetic variability of circulating strains in naturally infected cattle and how this correlates with serotyping and other standard tools currently used to characterize *Leptospira* strains.

### Data availability.

This whole-genome shotgun project has been deposited in DDBJ/ENA/GenBank under the BioProject numbers PRJNA543681 and PRJEB29877. Specifically, the data are available under the accession numbers ERS2975868, ERS2975869, ERS2975939, ERS2975870, ERS2975940, ERS2975942, ERS2975943, ERS2975946, ERS2975948, ERS2975949, ERS2976055, ERS2976056, ERS2976057, ERS2976058, ERS2976059, ERS2976060, ERS2976061, ERS2975938, ERS2975871, ERS2975941, ERS2976047, ERS2976048, ERS2976050, ERS2976052, ERS2976054, ERS2976062, ERS2975872, ERS2975944, ERS2975945, ERS2975947, ERS2976049, ERS2976051, ERS2976053, VCHF00000000, VCHG00000000, VCHH00000000, VCHI00000000, VCHJ00000000, VCHK00000000, and VCHL00000000.
